# rTMS as a Treatment of Alzheimer's Disease with and without Comorbidity of Depression: A Review

**DOI:** 10.1155/2013/679389

**Published:** 2013-02-07

**Authors:** Grant Rutherford, Rebecca Gole, Zahra Moussavi

**Affiliations:** ^1^Department of Electrical and Computer Engineering, University of Manitoba, Winnipeg, MB, Canada R3T 5V6; ^2^Graduate Program in Biomedical Engineering, Faculty of Engineering, University of Manitoba, Winnipeg, MB, Canada R3T 5V6; ^3^Riverview Health Center, 1 Morley Avenue, Winnipeg, MB, Canada R3L 2P4

## Abstract

With an ever-increasing population of Alzheimer's disease (AD) patients worldwide, a noninvasive treatment for AD is needed. In this paper, the application of repetitive transcranial magnetic stimulus (rTMS) as a treatment for patients with probable AD is compared to the application of rTMS as a treatment for depression. Comorbidity of depression and dementia is discussed, as well as possible links between the two diseases. The possible confounding antidepressant effects of rTMS on cognitive improvements in AD patients are discussed.

## 1. Introduction

In 2010, there were an estimated 35.6 million people in the world suffering from dementia [1]. This is an increasing problem; 65.7 million dementia cases are expected by 2030 and 115.4 million by 2050. The cause of 50%–75% of dementia cases is Alzheimer's disease (AD). This growing problem presents a pressing need for AD treatments. Repetitive transcranial magnetic stimulation (rTMS) is a noninvasive technique that has been used as a treatment for several major neurological and psychotic disorders such as Parkinson's disease, depression, and schizophrenia in the last decade [1]; it is currently being investigated as a treatment for AD.

The process of applying rTMS involves a rapidly varying magnetic field, which can be used to modulate the firing of neurons on the outer surface of the brain [2]. It has been shown that rTMS can either stimulate or inhibit cortical areas within the focal area of the coil [3, 4]. Often, rTMS treatments can be separated into two groups: high-frequency rTMS (10–20 Hz) and low-frequency rTMS (1 Hz) [4]. The high-frequency rTMS is generally considered to be more excitatory, while the low-frequency rTMS is inhibitory, although this may vary between individuals [5]. The mechanism through which rTMS affects the brain is thought to be long-term potentiation/depression (LTP/LTD) [6].

There have been a number of studies investigating rTMS as a treatment for cognitive decline in AD. A comprehensive review of these studies can be found in [7, 8]. These papers conclude that there is promise for the use of rTMS to treat AD, but that further study is required. Specifically, action and object naming has been shown to improve during rTMS treatments [9, 10], auditory sentence comprehension is improved up to eight weeks after rTMS treatment [11], high-frequency rTMS can improve cognitive skills in patients for up to 3 months [12], and rTMS combined with cognitive training produces long-lasting improvements in a variety of cognitive measures [13]. While these results are promising, they are still preliminary, and much further research is needed to confirm their results.

The purpose of this paper is to discuss the complications of comorbid depression on investigations of rTMS as a treatment for AD. The prevalence of comorbid depression among dementia patients has been reported as 19% in one study [14] and up to 68% in another [15]. While some previous studies explicitly excluded depressed patients [9–11], others did not [12, 13] perhaps due to lack of subjects excluding the comorbid syndrome. In order to properly understand the effects of rTMS on AD patients, it is important to consider the possibility that comorbid depression is also being treated.

## 2. Similarities in rTMS Protocols for Treatment of Dementia and Depression 

In the studies of rTMS treatments for AD patients, the dorsolateral prefrontal cortex (DLPFC) has been commonly used as the stimulation site [9–12], although at least one study also stimulated other brain regions [13]. Due to its interconnectedness with other brain regions, the DLPFC plays an important role in executive functions of the brain. This region coordinates functions with the rest of the brain, knows where specific information is stored, and can assess when information is needed. The DLPFC also helps to shift between tasks and therefore has a role in working memory. Further, it has a role in choice and decision-making. In early dementia, there are often problems with both working memory and adaptive decision-making, leading to the conclusion that the DLPFC is affected by dementia [16].

The most common application of rTMS clinically is for treatment of major depression. As with AD, the DLPFC is often targeted in the treatment of depression. This is because in depressed patients there is often a decrease in glucose uptake and blood flow in the left DLPFC, and by stimulating the left side or inhibiting the right side, the activity on both sides of the brain is equalized, producing antidepressant effects [17].

A review published in 2011 suggested the optimal specifications for the use of rTMS as a treatment for depression [18]. These specifications are very similar to those used in the rTMS studies to treat AD (see Table 2). The motor threshold intensities used to treat dementia vary from 90% to 110%, similar to the suggested 90%–120% intensity range for depression. The suggested frequency for depression treatment is 5–20 Hz, and frequencies of 10 Hz [13] and 20 Hz [9–12] have been used for dementia treatment. For depression, 5 treatments per week for 2–9 weeks are recommended [18]. The studies for dementia have implemented similar treatment schedules, including 5 sessions per week for one week [12], 2–4 weeks [11], and 6 weeks [13], occasionally with maintenance treatments [13] and follow-up assessments [11, 12]. Due to similar rTMS treatment protocols, antidepressant effects may be expected in dementia patients undergoing rTMS. For instance, in a comparative study of high-frequency and low-frequency rTMS for AD patients [12], high-frequency rTMS yielded significant improvements in the Geriatric Depression Scale, in addition to improvements in the Mini-Mental State Examination and Instrumental Daily Living Activity Scale.

In three of the five studies considered in this paper [9–11], patients with major depression were excluded, and therefore the positive effects were likely not caused by the antidepressant nature of the treatments. In a study that incorporated cognitive training [13], 6 of the 8 patients had comorbid depression. These cases were mild and controlled by medication, and 4 of these patients were in remission from their depression. A comparative study between high-frequency and low-frequency rTMS as a treatment for AD [12] did not specify the inclusion or exclusion of depressed patients, but the authors noted that approximately 40% of AD patients experienced depressive symptoms. Furthermore, one of the rating scales used for that study was the Geriatric Depression Scale (GDS), and the patients improved in the GDS as a result of treatments [12].

## 3. rTMS Study Protocols and Results

Five studies which apply rTMS to patients with Alzheimer's disease have been conducted [9–13] and are described below. The first studies [9, 10] considered the effects of rTMS on action and object naming, while the third [11] considered the effects on language production and comprehension. Another study [13] aimed to treat AD using rTMS combined with cognitive training, and the fifth study [12] investigated the cognitive improvements associated with rTMS.

In a study in 2006 [9], rTMS was applied to 15 patients with mild-to-moderate cognitive decline (probable dementia), and the effects were assessed by testing object and action naming during stimulation. Depressed patients were excluded from this study. The results showed that action naming performance was better during both left and right DLPFC stimulation compared to the sham stimulation. There was no significant increase in the performance seen for object naming. In a follow up study in 2008 by the same group, rTMS was applied to 24 AD patients: 12 with moderate-to-severe AD, and 12 with mild AD [10]. The experimental protocol was kept the same, and depressed patients were still excluded. The results showed that rTMS application to the DLPFC improved both action and object naming for moderate and severe AD patients but improved only action naming for mild AD patients.

The same group conducted a further study [11] to assess whether rTMS caused cognitive improvements in language production and/or comprehension in AD patients, and also to observe whether or not the improvements persisted after stimulation. Ten patients with probable moderate AD, and without major depression, were recruited and separated into two groups: one receiving 2 weeks of treatment and the other receiving 4 weeks of treatment. Subjects were assessed 8 weeks after treatment. The results showed a significant effect of rTMS treatment on auditory sentence comprehension. The improved naming performance demonstrated in the two previous studies was not observed. This result suggests that rTMS treatments improve naming performance only during treatments. The study also found that the patients with 4 weeks of treatment did not show significantly higher improvements than patients with 2 weeks of treatment. 

In a recent study [13], high-frequency rTMS was applied in conjunction with cognitive training (COG), with the goal of treating AD patients. This treatment (rTMS-COG) was applied to 8 patients with probable early or moderate AD. The trial consisted of daily treatments (5 days per week) for 6 weeks, followed by maintenance sessions (2 days per week) for 3 months. Six brain regions were stimulated: Broca and Wernicke, right and left DLPFC, and right and left parietal somatosensory association cortex (R-pSAC, L-pSAC). The brain areas were stimulated separately, 3 regions per day, every other day. The results showed a significant improvement in AD Assessment Scale-Cognitive (ADAS-cog) scores after the 6 weeks of treatments. After an additional 3 months of maintenance, the improvements were still evident. Only 7 patients completed the entire 4.5 months of the study. It should be noted that patients continued taking their prescribed medications during the trial. It should also be noted that 6 of the 8 patients had a history of depression, although 4 of these were in remission.

In another recent study, another group carried out a comparison of high-frequency (20 Hz) and low-frequency (1 Hz) rTMS in probable AD patients applied bilaterally over the DLPFC [12]. Forty-five patients diagnosed with probable AD (32 mild to moderate, 13 severe) were randomly separated into 3 groups: 15 patients received high-frequency treatments (20 Hz), 15 patients received low-frequency treatments (1 Hz), and 15 received sham treatments. Depressed patients were not explicitly excluded; however, measures of depression were evaluated during the study. Patients underwent daily rTMS sessions for 5 days. Assessments took place before and after the treatment plan, in addition to 1 month and 3 months after the last session. The results showed a significant improvement in the Mini-Mental State Examination (MMSE), Instrumental Daily Living Activity Scale (IADL), and Geriatric Depression Scale (GDS) for high-frequency treatments with moderate-to-mild AD patients. No such improvement was observed with severe AD patients. The high-frequency rTMS treatment yielded significantly more improvement in all rating scales compared to the low-frequency rTMS treatment and sham treatment. These improvements were still identified at the 3-month follow-up assessment.

A comparison summary of the rTMS protocols for the five aforementioned studies can be found in Table 1.

## 4. Comorbidity of Depression and Dementia

There is some concern that the beneficial results observed in AD patients undergoing rTMS treatments are due to the antidepressant effects of rTMS. As outlined in the previous section, the use of rTMS to treat depression is very similar to the protocol used to treat AD. Therefore, it is likely that for patients with comorbid depression the depression was treated during rTMS sessions. However, some of the aforementioned studies excluded patients with major depression [9–11], which may address this concern.

Several studies have considered the frequency of comorbid depression and dementia and the relationship between the two symptoms. A recent study conducted in Dutch psychogeriatric nursing home wards [14] found that, out of 496 psychogeriatric residents, 96 (or 19%) were found to have comorbid depression and dementia (Stages 2–6). Another study followed 670 outpatients with probable AD [19] and found that 26% of the patients had major depression, 26% had minor depression, and 48% were not depressed. A comparison of depression patients with reversible cognitive impairment (RCI) and with no cognitive impairment (NCI) [20] found that, within 5–7 years, 71.4% of the RCI group developed dementia, compared to only 18.2% of the NCI individuals. (RCI describes depressed patients who are first diagnosed with dementia but, upon treating for depression, show significant cognitive improvement [21].) This may suggest that patients with RCI are in a prodromal phase for dementia. The authors suggest that chronic depression may lead to dementia because of its effects on the brain (vascular effects, excessive release of corticosteroids, and hippocampal deterioration). Another study [22] following 1,239 older individuals (baseline age 55.5 ± 18.8 years) found that the risk of developing dementia increased by 87% for participants who had experienced 1 episode of elevated depressive symptoms (EDS) over the years of followup (median of 24.7 years), and nearly doubled for participants who had experienced 2 or more EDS. As noted by the authors, these results support the hypothesis that, in some cases of major depression, there is an excessive release of glucocorticoid (a type of corticosteroid), causing overstimulation of hippocampal glucocorticoid receptors, leading to the death of neurons in the hippocampus. Further, a study of 949 Framingham Heart Study participants [23] found that, over a 17-year follow-up period, 164 participants developed dementia (136 cases of AD). Among those depressed at baseline, 21.6% had developed dementia, compared to 16.6% of the participants who were not depressed at the baseline. These results suggest that depression may increase the risk of developing dementia. The authors propose that chronic inflammatory changes associated with depression may increase the likelihood of developing dementia and AD. Vascular factors may also play a role, as well as lifestyle factors associated with depression.

## 5. Conclusion

From the five studies that have applied rTMS to AD patients, it seems clear that high-frequency rTMS applied to the DLPFC presents a possible treatment tool for AD. More studies should be conducted with larger populations to verify the results of these studies, and take into consideration the antidepressant effects of rTMS and how they may be causing the observed cognitive improvements. In particular, care should be taken in all future studies to separate the results from patients with depression or a history of depression from other results. This will ensure that any confounding factors relating to cognitive improvements from the treatment of depression are fully understood.

In addition, it seems likely that some cases of depression may lead to dementia later in life. Depression with reversible cognitive impairment may be a prodromal phase for dementia. Positive results have been found using rTMS as a treatment for both depression and dementia patients with similar application techniques. Therefore, treating dementia patients with rTMS may not only improve cognitive abilities, but also have antidepressant effects for those who are suffering from comorbid depression. In addition, it is possible that by also treating the depressive symptoms of dementia patients, further damage to the brain from depressive effects could be slowed. In order for these exciting possibilities to be explored, we should ensure that we fully understand both the cognitive and antidepressant potential of rTMS.

## Figures and Tables

**Table 1:** Comparison of rTMS protocols for treatment of Alzheimer's disease.

Paper title	Number of participants	Depression	Participant age (Mean ± SD)	Level of dementia (AD)	Stimulation site	Frequency	Intensity	Length of trial
Effect of transcranial magnetic stimulation on action naming in patients With Alzheimer's disease [9]	15	Excluded	76.6 ± 6	Mild-moderate cognitive decline, probable AD	Right and left DLPFC, sham	20 Hz	90%	

Transcranial magnetic stimulation improves naming in Alzheimer's disease patients at different stages of cognitive decline [10]	24	Excluded	Moderate-severe AD: 77.6 ± 5.8 Mild AD: 75 ± 6.2	12 moderate-severe AD, 12 mild AD	Right and left DLPFC, sham	20 Hz	90%	

Improved language performance in Alzheimer's disease following brain stimulation [11]	10 (5 real-real stimulation, 5 placebo-real stimulation)	Excluded	74.4 ± 3.8	Probable moderate AD	Left DLPFC	20 Hz	100%	2 weeks (5/week) real or placebo, followed by 2 weeks all real

Beneficial effect of repetitive transcranial magnetic stimulation combined with cognitive training for the treatment of Alzheimer's disease: a proof of concept study [13]	8	6/8 had history of depression	75.4 ± 4.4	Probable early-moderate AD (Clinical Dementia Rating of 1)	6 regions (3 per day, every other day) (i) Broca, Wernicke and right DLPFC (ii) left DLPFC and right and left parietal somatosensory association cortex (pSAC)	10 Hz	90% for Broca and DLPFC, 110% for Wernicke and pSAC	6 weeks (5/week), followed by maintenance (2/week) for 3 months

Effects of low versus high frequencies of repetitive transcranial magnetic stimulation and functional excitability in Alzheimer's dementia [12]	45 (15 HF stimulation, 15 LF stimulation, 15 sham stimulation)	Depression not excluded. Assessment of patient depression improved with rTMS.	68.4 (SD not specified)	32 probable mild-moderate AD, 13 probable severe AD	Right and left DLPFC (right, then left hemisphere consecutively stimulated)	HF: 20 Hz LF: 1 Hz	HF: 90% LF: 100%	5 days

**Table 2:** Comparison of rTMS applied to depression and Alzheimer's disease.

	Common stimulation site	Frequency	Intensity of motor threshold	Sessions per week	Number of weeks
Depression	DLPFC (often left)	5–20 Hz	90%–120%	5	2–9
Alzheimer's Disease	DLPFC (left or bilateral), Broca, Wernicke, and pSAC	10 Hz, 20 Hz	90%–110%	5	1–6
